# Draft Genome Sequence of *Bacillus amyloliquefaciens* Strain RHNK22, Isolated from Rhizosphere with Biosurfactant (Surfactin, Iturin, and Fengycin) and Antifungal Activity

**DOI:** 10.1128/genomeA.01682-15

**Published:** 2016-01-28

**Authors:** Papathoti Narendra Kumar, T. H. Swapna, Koppula Sathi Reddy, K. Archana, Lingampalli Nageshwar, S. Nalini, Mohamed Yahya Khan, Bee Hameeda

**Affiliations:** Department of Microbiology, University College of Science, Osmania University, Hyderabad, India

## Abstract

*Bacillus amyloliquefaciens* strain RHNK22 isolated from groundnut rhizosphere showed direct and indirect plant growth-promoting traits along with biosurfactant activity and reduction in surface tension of water. Biosurfactants were identified as lipopeptides (surfactin, iturin, and fengycin) by molecular and biochemical analysis in our studies.

## GENOME ANNOUNCEMENT

*Bacillus amyloliquefaciens* strain RHNK22 was isolated from groundnut by the enrichment culture method as described by Dubey and Juwarkar (1). *B. amyloliquefaciens* RHNK22 has been used to produce industrially important biosurfactants with surface tension reduction and antifungal activity (2), plant growth-promoting traits, and antioxidant and probiotic properties (unpublished data) and was thus subjected to genomic sequencing.

The shotgun sequencing of *Bacillus amyloliquefaciens* strain RHNK22 genome was performed using a paired-end (PE) 2 × 150 bp library on the Illumina platform (NextSeq 500). The draft genome, as submitted to GenBank, was 3,978,182 bp in length with a mean scaffold size of 1,037,178 bp, a median size of 48,109 bp, and a maximum length of 987,166 bp. The mean G+C content of the genome was found to be 46.02% using the Velvet *de novo* assembler (3). Coding DNA sequence (CDS) prediction was performed using Prodigal v2.60. A total of 3,872 CDS were identified from the *B. amyloliquefaciens* strain RHNK22 assembly. BLASTx analysis was performed using NCBI BLAST+ to search 3,872 CDS against the nonredundant (NR) database.

A significant *E* value of 10^−5^ was used as a filter to retain significant BLAST hits and remove short ambiguous alignments. The top BLAST result distributions were obtained by calculating the total number of CDS for each reported species (4). A total of 3,872 CDS were annotated with NCBI BLAST. A total of 3,611 out of 3,872 CDS represent homologs in *B. amyloliquefaciens. B. amyloliquefaciens* sp. *plantarum* CAUB946 is the most predominant strain among *B. amyloliquefaciens* species in the BLAST result which is used for single nucleotide polymorphism (SNP) analysis. SNP discovery was carried out using the draft genome identified through BLAST. The reads of the samples were mapped on the reference genome (NC_016784) using Bowtie 2. A total of 16,264 SNP were identified after filtration for a read depth of 10 and quality score of 20. The quality filtered SNP were filtered for indels (5). A total of 15,701 SNP were identified after indel filtration with 89.99%overall rapid mapping. Subsequently, SNP were discarded by taking a flanking distance of 100 bp and we obtained 7,986 SNP.

Gene ontology (GO) assignments were performed to classify the functions of the CDS based on gene ontology terms (6). GO mapping will provide the ontology of defined terms representing gene product properties which are grouped into three main domains: biological process, molecular function, and cellular component. The GO mapping was performed using the BLAST2GO program using a genus database generated from *B. amyloliquefaciens* sp. genomes, *B*. *amyloliquefaciens* sp. *plantarum* CAUB946 (GenBank accession number NC_016784), *B. amyloliquefaciens* FZB42 (GenBank accession number NC_009725), and *B. amyloliquefaciens plantarum* UCMB 5036 (GenBank accession number NC_020410). The GO annotation results of *B. amyloliquefaciens* strain RHNK22 were classified into 2,456 molecular function GO-terms, 2,572 GO-terms classified as biological processes, and 1,063 GO-terms in the cellular component category. The tRNA scan SE program was used to predict the 79 t-RNA in *B. amyloliquefaciens* strain RHNK22. A total of 5 r-RNA were identified in the *B. amyloliquefaciens* strain RHNK 22 sample using the RNAmmer 1.2 server.

### Nucleotide sequence accession number.

This whole-genome shotgun project has been deposited in NCBI GenBank under the accession number LMAG00000000.
